# Artificial Intelligence in Colorectal Cancer Screening, Diagnosis and Treatment. A New Era

**DOI:** 10.3390/curroncol28030149

**Published:** 2021-04-23

**Authors:** Athanasia Mitsala, Christos Tsalikidis, Michail Pitiakoudis, Constantinos Simopoulos, Alexandra K. Tsaroucha

**Affiliations:** 1 Second Department of Surgery, University General Hospital of Alexandroupolis, Democritus University of Thrace Medical School, Dragana, 68100 Alexandroupolis, Greece; ctsaliki@med.duth.gr (C.T.); pterion_ts@yahoo.gr (M.P.); cksimop@gmail.com (C.S.); 2 Laboratory of Experimental Surgery & Surgical Research, Democritus University of Thrace Medical School, Dragana, 68100 Alexandroupolis, Greece; aktsarou@gmail.com

**Keywords:** artificial intelligence, colorectal cancer, colonoscopy, screening, computer-aided detection, computer-aided diagnosis, robotic-assisted surgery, therapy, precision oncology

## Abstract

The development of artificial intelligence (AI) algorithms has permeated the medical field with great success. The widespread use of AI technology in diagnosing and treating several types of cancer, especially colorectal cancer (CRC), is now attracting substantial attention. CRC, which represents the third most commonly diagnosed malignancy in both men and women, is considered a leading cause of cancer-related deaths globally. Our review herein aims to provide in-depth knowledge and analysis of the AI applications in CRC screening, diagnosis, and treatment based on current literature. We also explore the role of recent advances in AI systems regarding medical diagnosis and therapy, with several promising results. CRC is a highly preventable disease, and AI-assisted techniques in routine screening represent a pivotal step in declining incidence rates of this malignancy. So far, computer-aided detection and characterization systems have been developed to increase the detection rate of adenomas. Furthermore, CRC treatment enters a new era with robotic surgery and novel computer-assisted drug delivery techniques. At the same time, healthcare is rapidly moving toward precision or personalized medicine. Machine learning models have the potential to contribute to individual-based cancer care and transform the future of medicine.

## 1. Introduction

Artificial intelligence (AI) is the field of computer sciences devoted to building smart machines capable of performing tasks that typically require human-level intelligence [1]. Several AI applications are all around us, yet it can be hard to understand and evaluate their impact on today’s society. Over the last decade, the significant contribution of deep learning techniques and support vector machines (SVMs) to this advanced technology has played a critical role in medicine and healthcare systems.

**Figure 1:**
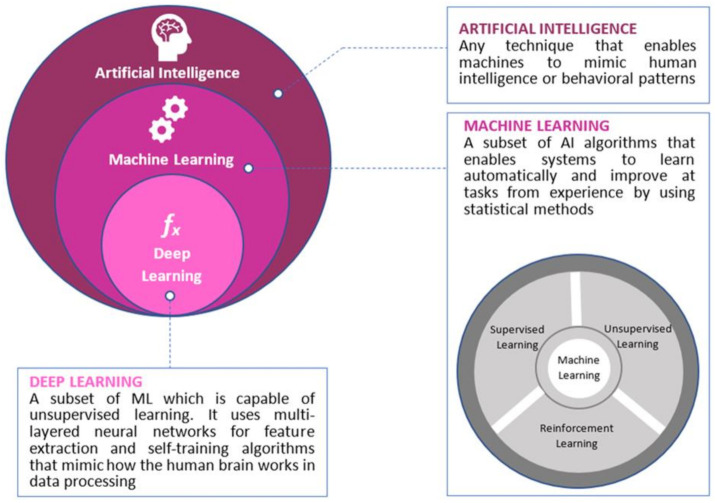
An overview of the differences between artificial intelligence (AI), machine learning (ML), and deep learning (DL).

In general, AI applications in the medical field have two main branches: virtual and physical. Machine learning (ML) and deep learning (DL, a subset of ML) constitute the virtual component of AI [2]. ML algorithms are further classified into supervised, unsupervised and reinforcement learning (Figure 1). Meanwhile, the most important deep learning scheme, a convolutional neural network (CNN), represents a particular type of multilayer artificial neural network that is highly efficient for image classification [3]. To date, the progress in the development of neural network models has permeated the field of medicine with great success [3]. In addition to the virtual part, the physical branch of AI includes medical devices and robots, such as the da Vinci Surgical System (Intuitive Surgical Inc., Sunnyvale, CA, USA) and nanorobots for targeted drug delivery [2].

This novel technology has made unique contributions to the diagnosis and therapy of several types of cancer, including colorectal carcinomas. With the intent of improving the screening, diagnosis, and treatment strategies for colorectal cancer (CRC) patients, current studies have shown that AI-guided care can play a pivotal role in clinical practice [4,5]. Recently, researchers designed AI models to reduce the rates of missed adenomas and the risk of developing cancer by improving CRC screening outcomes [5,6]. Computer-aided detection and characterization systems are now attracting increased interest and attention. The AI assistance for colorectal polyp detection and optical diagnosis in colonoscopy may help endoscopists make accurate and timely diagnoses [5].

Furthermore, CRC treatment enters a new era with robotic surgery and novel computer-assisted drug-delivery techniques. At the same time, healthcare is rapidly moving toward precision or personalized medicine [7]. Machine learning models have the potential to contribute to individual-based cancer care and transform the future of medicine. Our review herein aims to provide in-depth knowledge and analysis of the AI applications in CRC screening, diagnosis, and treatment based on current literature. In addition, we explore the role of recent advances in AI systems regarding medical diagnosis and therapy, with several promising results.

## 2. Artificial Intelligence, Colorectal Cancer and Genomics

Overall, integrating AI algorithms with genetic testing has shown several promising results for CRC. Based on gene expression, Hu et al. [8] performed a simulation experiment to classify 53 colon cancer patients with the Union for International Cancer Control (UICC) II into two groups: relapse and no relapse after surgery. The researchers compared the classification accuracy obtained by the S-Kohonen (91%), Back-propagation (BP, 66%), and SVM (70%) neural networks. They suggested that the S-Kohonen neural network is more effective for colon cancer classification. Based on SVM analyses, Xu et al. [9] conducted a study to identify differentially expressed genes (DEGs) to distinguish patients with a high recurrence risk of colon cancer. Interestingly, they identified a 15-gene signature that may be useful as a predictor of recurrence risk and prognosis for colon cancer patients. In 2019, Zhang et al. [10] proposed a sensitive and low-cost method for detecting the B-rapidly accelerated fibrosarcoma (BRAF) gene mutation, which involves a substitution of valine to glutamic acid at codon 600 (V600E). When used to test for the v-raf murine sarcoma viral oncogene homolog B1 (BRAF) V600E mutation in colorectal carcinomas, the current model demonstrated 100% diagnostic sensitivity, 87.5% diagnostic specificity and 93.8% diagnostic accuracy. This novel approach, which was based on near-infrared (NIR) spectroscopy in conjunction with counter propagation artificial neural network (CP-ANN), can also help distinguish between the BRAF V600E mutant and the wild type.

In 2015, a research group designed artificial neural networks to investigate the correlation between genetic and environmental factors to DNA methylation in CRC [11]. A few years later, Wang et al. [12] combined gene expression profiling data from The Cancer Genome Atlas (TCGA) database and analysis with AI algorithms to improve CRC diagnosis. They used BP and learning vector quantization (LVQ) neural networks to build four diagnostic models; Cancer/Normal, M0/M1, carcinoembryonic antigen (CEA) testing (<5/≥5), and clinical staging (I–II/III–IV). The predictive accuracy and area under the curve of the Cancer/Normal, M0/M1, CEA and Clinical stage models were 100%, 1.000; 87.14%, 0.670; 100%, 1.000; and 100%, 1.000, respectively. Furthermore, Wan et al. [13] proposed a machine learning method using tumour-derived cell-free DNA that resulted in high sensitivity and specificity. Their technique may constitute a promising future direction for further research in early-stage CRC detection. In another study, Kel et al. [14] designed a method referred to as “walking pathways” to identify potential methylated DNA biomarkers and then applied AI techniques in order to analyze cancer-specific enhancers.

Increasing evidence shows that non-coding RNAs (ncRNAs) contribute to every stage of colorectal tumorigenesis and cancer progression by influencing essential signaling pathways, including WNT/β-catenin, phosphoinositide-3-kinase (PI3K)/ protein kinase B (Akt), epidermal growth factor receptor (EGFR), NOTCH1, mechanistic target of rapamycin (mTOR) and TP53 [15]. As current studies shed light on ncRNAs’ role in cancer diagnosis and treatment, identifying specific ncRNA expression alterations in colon tissue and plasma/serum samples may prove valuable in early CRC diagnosis, prognosis prediction and targeted therapy. Chang et al. [16] used artificial neural network analysis to compare the expression profiles of 380 micro RNAs (miRNAs) in stage II colorectal tumours and normal tissues. They identified a 3-miRNA signature (miR-139-5p, miR-3, and miR-17-92) for predicting the tumour status in stage II CRC. With the intent of optimizing this method and improving the prediction accuracy, a team of researchers designed a novel computational algorithm for miRNA-target prediction in colorectal carcinomas using a Naive Bayes classifier [17]. The current model, which was named as CRCmiRTar, was also able to unravel the CRC-specific interactions between miRNAs and target messenger RNAs (mRNAs). In fact, identifying disease-specific miRNA target interactions may significantly contribute to the development of potential drug targets.

In another study, a research group from Spain evaluated the performance of a 6-miRNA signature (miRNA19a, miRNA19b, miRNA15b, miRNA29a, miRNA335, and miRNA18a) in plasma samples using a robust predictive model for the differentiation between healthy individuals and patients with CRC and advanced adenomas [18]. The SVM classification model demonstrated 85% sensitivity and 90% specificity. Afshar et al. [19] proposed an artificial neural network model, which accurately classified the sample data into cancerous and non-cancerous by screening four CRC-specific miRNAs retrieved from the Gene Expression Omnibus (GEO) database. In addition, Xuan et al. [20] suggested using a dual CNN prediction model for discovering potential disease-related miRNAs. Their AI-based system, CNNDMP, explores the deep features of miRNA similarities, the disease similarities, and the miRNA-disease associations. Case studies on breast, colorectal and lung cancer confirm the powerful abilities of the CNNDMP for identifying potential disease-related miRNAs.

To date, AI systems enable clinicians to predict the prognoses of patients with CRC. Based on various machine learning methods, Gründner et al. [21] used gene markers to train predictive models for measuring disease-free survival, overall survival, radio-chemotherapy response and relapse. In another study, the researchers detected 40 SVM-classified signature genes in metastatic colorectal neoplasms, as well as adenosine monophosphate (AMP)-activated protein kinase (AMPK) signaling and ubiquitin-mediated proteolysis pathways [22]. These genes may be utilized as biomarkers for the prognosis of metastatic CRC. Despite the lack of experimental validation, the current model can precisely distinguish the metastatic CRC samples from the non-metastatic ones. Recently, Ge et al. [23] conducted a study to investigate the role of immune cells and immune-related gene expression in the surrounding tumour microenvironment of CRC. They used a deconvolution algorithm, CIBERSORT, to analyze the infiltration of 22 immune cell types in the tumour microenvironment and immune-related gene expression in 404 CRC and 40 adjacent non-tumorous tissues. In general, their findings may eventually help clinicians accurately select the targets for immunotherapies and individualize strategies for managing CRC patients.

## 3. Colorectal Cancer Screening

CRC represents the third most commonly diagnosed malignancy in both men and women [24]. It is responsible for a significant increase in the estimated number of cancer-related deaths worldwide [24]. Approximately 60–70% of CRC patients with clinical manifestations are diagnosed at advanced stages of the disease [25]. However, early-stage detection may improve the patients’ clinical outcomes in terms of avoiding delays in treatment and reducing CRC morbidity and mortality [26].

CRC is a highly preventable disease, and routine screening appears to be an important step in declining the incidence rates of this malignancy [27]. The alterations from normal mucosa to a premalignant growth and then to a malignant lesion take almost 15 to 20 years. The polyp–cancer sequence evolves slowly and may eventually take 10 or more years for colorectal polyps to transform into malignant structures [28].

Effective screening methods have been developed to identify abnormal tissue, which may be indicative of either a premalignant precursor lesion or an early-stage tumour [29,30,31]. Available modalities for CRC screening include invasive (colonoscopy and flexible sigmoidoscopy) and minimally invasive (capsule endoscopy) techniques, imaging examinations (computed tomographic colonography), blood and stool tests, such as guaiac fecal occult blood test (FOBT), fecal immunochemical test (FIT), and multitarget stool DNA (MT-sDNA) test [32,33]. Machine learning algorithms may be utilized as non-invasive and cost-effective methods to screen the CRC risk in large populations using personal health data [34].

### 3.1. Colonoscopy

The detection and resection of precancerous lesions during a colonoscopy are of utmost importance to reduce the risk of developing CRC. Several studies have shown that even though a colonoscopy is considered the “gold-standard” screening test, it is not perfect [35]. Indeed, it is worth mentioning at this point that interval cancer may be occasionally detected in patients with a previously negative colonoscopy [35]. Interval colorectal cancer is defined as a primary cancer diagnosed after a negative scheduled screening test within a time period equal to the screening interval. In fact, previous research revealed that 8.6% of cases with CRC occur within three years following a colonoscopy, which has yielded a negative result [36].

High adenoma detection rate (ADR) is inversely correlated with adenoma miss rate (AMR) and the risk of post-colonoscopy CRC [37,38]. Corley et al. demonstrated that every 1% increase in ADR is associated with a 3% reduction in the risk of CRC development and a 5% reduction in the risk of fatal CRC [38]. Overall, ADRs may range from 7% to 53% between different endoscopists [38]. During the procedure, AMRs also vary greatly between 6–27% depending on several factors [39]. Current evidence reveals that these factors include the quality of preprocedural bowel preparation, time of withdrawal, operator experience and training, use of sedation, cecal intubation rate, visualization of flexures (blind spots), use of image-enhanced endoscopy and presence of flat or diminutive (≤5 mm) and small (<10 mm but >5 mm) polyps [38,40,41,42]. Regarding the size of lesions, a systematic review and meta-analysis showed that the total AMRs for adenomas between 1–5 mm, 5–10 mm, and larger than 10 mm were 26%, 13% and 2.1%, respectively [43].

**Figure 2:**
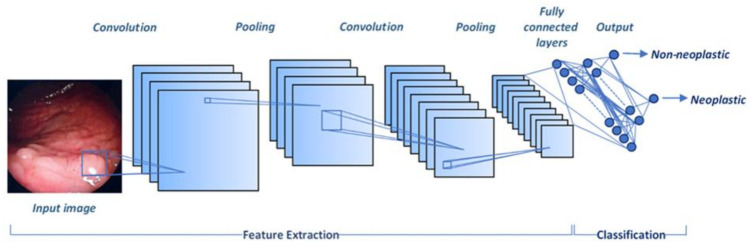
A convolutional neural network (CNN) design for colorectal polyp classification. CNN is a multilayer artificial neural network typically composed of three types of layers; convolution, pooling, and fully connected layers. Feature extraction from an input image is performed from the first two layers. The fully connected layers are used to map these features into a final output. CNN, convolutional neural network.

Furthermore, several studies suggest that ADRs may be increased up to 30–50% with an additional observer*’*s contribution to patients undergoing screening colonoscopy [44,45]. In view of the high risk of developing CRC, the assistance of real-time automatic polyp detection systems could significantly reduce missed diagnosis rates and help clinicians detect polyps in real-time. This subject represents an area of particular and growing interest in the field of AI-assisted colonoscopy, along with the recent advancements in technology and modern science. To date, technological progress is considered a necessary step in minimizing the risk of missed diagnosis and helping endoscopists to visualize and evaluate precancerous polyps. In fact, the significant role of computer-aided detection (CADe) and diagnosis (CADx) systems during colonoscopy in automated polyp detection and further characterization is recently discovered [5]. Current novel technologies have been applied in order to aid adenoma detection by using deep learning techniques. With the intent of improving ADRs, computer algorithms driven by CNNs may accurately detect and localize the presence of premalignant lesions [46]. A CNN represents a particular type of artificial neural network and deep learning technique that is highly effective at performing medical image analysis [47] (Figure 2).

Recently in the first prospective randomized controlled trial, Wang et al. [48] studied the effect of a deep learning-based CADe model on polyp and adenoma detection rates. Out of 1058 patients, 536 were randomized to a conventional colonoscopy, and 522 were randomized to a colonoscopy with a computer-aided detection (CADe) system. Hollow blue tracking boxes appeared on the screen to highlight the specific region of interest and show the detected by algorithm polyps. In the CADe group, the results obtained revealed an increase in both ADR (29.1% vs. 20.3%, *p* < 0.001) and the mean number of identified adenomas per patient (0.53 vs. 0.31, *p* < 0.001), when compared with the standard colonoscopy group. Interestingly, the high ADRs of the present automated polyp detection system were attributed to the detection of a large number of diminutive polyps (185 vs. 102, *p* < 0.001). The results of this study also revealed that there was no statistical difference in the detection of large adenomas between the two groups (77 vs. 58, *p* = 0.075), but a major increase in the number of detected hyperplastic polyps in the CADe group was noted (114 vs. 52, *p* < 0.001). Indeed, the impact of AI-assisted colonoscopy on identifying small polyps, which could eventually be missed even by highly skilled endoscopists, is widely realized. Even though there is a tendency to underestimate the correlation between malignancy and the small size of polyps, high detection rates in diminutive polyps may lower the risk of interval CRC. Mori et al. [49] demonstrated that CADx assistance during a colonoscopy might also help endoscopists distinguish neoplastic from non-neoplastic polyps, leading to a “diagnose-and-leave” strategy for the last ones.

### 3.2. Virtual Colonoscopy

Virtual colonoscopy or computed tomographic colonography (CTC) is a modified computed tomography (CT) examination, which was first described in 1994 by Vining et al. [50] and represents an alternative screening tool to conventional colonoscopy for CRC patients. With the intent of improving colorectal polyp detection and classification, AI-based algorithms may provide computer-aided solutions to achieve optimal diagnostic performance and image quality in CTC. According to the Haralick texture analysis method, Song et al. [51] presented a virtual pathological model to explore the usefulness of high-order differentiations, including gradient and curvature. The results of this research revealed that the area under the receiver operating characteristic (ROC) curve (AUC) of classification in distinguishing colorectal lesions (neoplastic and non-neoplastic) was improved from 0.74 (by using the image intensity solely) to 0.85 (by also using the texture features from high-order differentiations). In another study, Grosu et al. [52] developed a machine learning method to distinguish between benign and precancerous CTC-detected colorectal polyps in an average-risk asymptomatic CRC screening sample. The current classification algorithm showed promising results with a sensitivity of 82%, a specificity of 85%, and an AUC of 0.91.

Meanwhile, AI may also contribute to other challenging issues, such as the automatic detection of flat neoplastic lesions, eventually reducing the interval cancer risk. According to the Paris classification, adenomas may present as either protruding (pedunculated and sessile) or non-protruding (elevated, flat and depressed) lesions [53,54]. In fact, the presence of flat colorectal adenomas may represent an aggressive pathway in tumorigenesis and a determining factor in increased AMRs [55]. In previous research, Taylor et al. [56] designed a CADe model to examine its diagnostic capability for flat early-stage CRC (T1) using CTC. The CADe system, which was applied at three settings of sphericity, revealed that there was noted an inverse correlation between adenoma detection sensitivity and sphericity (83.3%, 70.8% and 54.1% at sphericity of 0, 0.75, and 1, respectively), and a direct correlation between accuracy and sphericity. Hence, the current study indicates that novel applications of computer-aided systems through CTC may effectively detect even flat CRC.

### 3.3. Capsule Endoscopy (CE)

CE is a minimally invasive technique, usually well tolerated by the patients, which can be used as an alternative approach for CRC screening, especially in incomplete colonoscopy cases. Colon capsule endoscopy is generally based on the use of laxatives and requires manual interpretation and analysis of the acquired images for colorectal lesion detection. Overall, the capsule moves through the gastrointestinal (GI) tract depending on intestinal motility. However, CE prolonged reading time, which may take about 45 min, can be particularly time-consuming [57,58]. It is worth noting that AI-based techniques may lead to high ADRs by automating the reading and examination of the results and reducing the associated risk of human error [59,60]. In a research work conducted by Blanes-Vidal et al. [59], a novel algorithm was developed to match CE and colonoscopy-identified polyps based on three variables: their estimated size, location and morphology. Another algorithm was also proposed in this study based on a deep convolutional neural network for automatic colorectal polyp detection and localization [59]. The current innovative AI-assisted model resulted in high sensitivity (97.1%), specificity (93.3%), and accuracy (96.4%) for identifying polyps when compared with the manual process of polyp detection.

### 3.4. Blood Tests

Furthermore, the impact of AI-assisted techniques on blood tests to identify early-stage CRC is thoroughly examined. Regarding blood fluorescence spectroscopy, Soares et al. [61] proposed a classification model composed of a binary SVM (first level) and a one-class SVM (second level) classifier. At the first level, CRC samples were differentiated from normal samples (87% sensitivity, 95% specificity). As for non-CRC samples, at the second level, there were either non-malignant lesions or no findings at all (60% sensitivity, 79% specificity).

In general, blood test results and demographic characteristics may be used to evaluate a person*’*s risk of developing CRC. Information from a complete blood count (CBC), including findings indicative of either microcytic iron deficiency anemia or a combination of anemia and elevated red cell distribution width (RDW), may help physicians estimate the cancer risk [62,63,64]. In fact, previous research revealed that RDW showed a sensitivity of 84% and a specificity of 88% for right-sided colon cancer [46]. In a binational retrospective study, Kinar et al. [63] used electronic medical records of two independent (unrelated) groups of individuals (Israeli and UK datasets) and designed an AI-assisted prediction model (MeScore*^®^*, Calgary, Alberta, Canada) for identifying people at high risk for CRC. Taking into account a few parameters (age, sex, and CBC data collected 3 to 6 months prior to the cancer diagnosis), there were observed comparable results between these two different populations. Indeed, the results obtained (for the Israeli and UK validation sets, respectively: AUC for CRC detection was 0.82 ± 0.01 and 0.81, specificity at 50% sensitivity was 88 ± 2% and 94 ± 1%) suggest that the current risk prediction model should be generally applied in other groups as well in order to identify those individuals requiring further clinical evaluation and screening. The present study revealed that the combination of this model and FOBT contributed to a 2.1-fold increase in cancer detection in the Israeli dataset. Moreover, Kinar et al. [65] proposed using additional markers to improve the accuracy of the CRC risk prediction algorithm.

In a current study [66], a machine learning-based algorithm that incorporates basic patient characteristics and demographic data with CBC test results was evaluated to identify patients at increased risk for CRC that may benefit from more intensive screening. The ColonFlag*^®^* software uses age, sex, and CBC information, including inflammatory cells, platelets, and red blood cell parameters, to generate a risk score for every individual. This AI application in routine blood tests is considered a passive test and a valuable option in identifying high-risk patients for CRC development, especially when the score is above the defined threshold.

The process of detecting and isolating circulating tumour cells in peripheral blood samples, could also be used as a novel method for CRC detection. In a cohort study of 47 subjects, the CellMax (CMx*^®^*) platform, an AI system based on the aforementioned procedure, achieved clinical sensitivity and specificity of 80% [67]. In addition, based on machine learning techniques, a current research work reported that AI applications could assist in analyzing the content of specific serum protein biomarkers, including leucine-rich alpha-2-glycoprotein 1 (LRG1), epidermal growth factor receptor (EGFR), inter-alpha-trypsin inhibitor heavy-chain family member 4 (ITIH4), hemopexin (HPX) and superoxide dismutase 3 (SOD3) in order to identify CRC with 70% sensitivity at over 89% specificity (AUC = 0.86) [68]. To date, blood-based screening approaches have been developed in an effort to detect the tumour at early stages.

## 4. Polyp Detection

Even though the colonoscopy is widely accepted as the “gold-standard” of CRC screening methods, it is worth mentioning that the current procedure is not 100% sensitive. Several factors, such as suboptimal bowel preparation, morphology and small size of polyps, are considered to affect the sensitivity of the colonoscopy. Using colonoscopy and histopathological data from the Netherlands cancer registry between 2001 and 2010, a retrospective population-based study revealed that 86.4% of post-colonoscopy CRC cases were associated with inadequate colon examination and missed or incompletely resected lesions during the previous colonoscopy examination [69]. The proportion of missed post-colonoscopy CRCs is higher for the patients with right-sided, flat or small polyps [36,69]. Currently, innovative software models are developed to improve ADR and, therefore, lead to the prevention of interval CRC [70,71]. A summary of recent studies on AI systems for colorectal polyp detection is presented in Table 1 [48,71,72,73,74,75,76,77,78,79,80,81,82,83,84,85,86,87].

Karkanis et al. [88] designed a CADe algorithm for polyp identification using color and texture analysis of the mucosal surface based on color wavelet covariance (CWC) features. By examining a dataset of 180 frame images extracted from 60 colonoscopic video sequences containing small polyps, the current method demonstrated a sensitivity and a specificity of 99.3 ± 0.3% and 93.6 ± 0.8%, respectively, for automatic colorectal polyp detection. However, this CADe software was able to identify precancerous lesions in static endoscopic images rather than being a real-time polyp recognition system during ongoing colonoscopy.

In 2016, Fernández-Esparrach et al. [71] developed and tested a model for using Window Median Depth of Valleys Accumulation (WM-DOVA) energy maps, which defined the presence of polyps and their boundaries, on 24 colonoscopy videos with 31 different polyps. However, this method yielded only 70.4% sensitivity and 72.4% specificity for polyp detection. In fact, the presence of vessels, stools, and folds gave rise to some false-positive results.

A deep learning detection model was proposed by Urban et al. [77] in order to evaluate the ability of a computer-aided image analysis system based on CNNs to identify polyps. In this work, a set of 8641 images with a total number of 4088 polyps derived from more than 2000 colonoscopy videos was utilized for training the current deep CNN. The CADe model detected polyps with an AUC of 0.991 and a cross-validation accuracy of 96.4%. In the analysis of nine randomly selected colonoscopy videos (*n* = 28 resected polyps), four expert reviewers detected eight additional polyps without the present CADe assistance (*n* = 36) and an additional seventeen polyps with CADe assistance (*n* = 45). This AI-based model showed a false positive rate of 7%.

Misawa et al. [76] designed a CADe system and evaluated its performance using a sample of 73 videos with 155 different polyps. Their technique displayed promising results in lowering AMRs, particularly when applied to small polyps. Frame-based (90% sensitivity, 63.3% specificity, and 76.5% accuracy) and polyp-based (94% sensitivity and 60% false-positive rate for polyp detection) analyses were conducted in the current study.

Another interesting research was performed in 2018 by Wang et al. [89] for automated polyp detection. A deep learning algorithm for colorectal polyp detection during colonoscopy was developed using a sample of 5545 images obtained from 1290 patients. Their method was evaluated based on four independent datasets. The researchers used Dataset A (27,113 static images from 1138 patients during a colonoscopy with at least one identified colorectal polyp showing per-image sensitivity, specificity, and AUC of 94.38%, 95.92%, and 0.984, respectively) and Dataset B (a public database of 612 images extracted from 29 video sequences showing per-mage sensitivity of 88.24%) for colonoscopy image analysis. They also used both Dataset C (a collection of 138 videos with histologically confirmed polyps from 110 patients with a per-image and per-polyp sensitivity of 91.64% and 100%, respectively) and Dataset D (54 unaltered videos without polyps with a per-mage specificity of 95.4%) for colonoscopy video analysis. This technique significantly contributed to real-time colonoscopy video analysis via processing at least 25 frames/second with a latency of 76.80 ± 5.60 milliseconds.

In general, a colonoscopy is an operator-dependent procedure, even though it remains the “gold-standard” procedure for polyp identification and localization. Therefore, one of the main questions that remains to be answered is whether challenges associated with the camera’s viewpoint, light conditions, size and morphology of colorectal polyps during routine colonoscopy may be overcome with the contribution of CADe systems. Indeed, AI-assisted models may be used as an «extra pair of eyes» and improve ADRs, especially with real-time computer-aided colonoscopy. However, further data collection and research are required to evaluate the performance of CADe systems in automatic polyp detection.

## 5. Polyp Characterization

In addition to polyp detection, computer-aided systems have shown great potential to improve the accuracy of histology prediction and characterization of colorectal polyps during a colonoscopy examination. In fact, an AI-based model may act as a virtual assistant to the endoscopists by enhancing their learning phase, differentiating neoplastic lesions from non-neoplastic mimickers, and predicting the presence or even the depth of submucosal invasion in patients with colorectal carcinomas [90,91]. Artificial intelligence for real-time optical diagnosis may also help determine the most appropriate treatment approach and avoid any unnecessary polypectomies or their associated post-procedural complications. With the intent of directing the rapid endoscopic technology development, the Preservation and Incorporation of Valuable endoscopic Innovations (PIVI) initiative (an American Society for Gastrointestinal Endoscopy (ASGE) program) suggested that a novel technology should achieve a threshold of negative predictive value (NPV) >90% for the optical diagnosis of diminutive colorectal polyps [92]. A summary of recent studies on AI systems for colorectal polyp characterization is presented in Table 2 [49,83,93,94,95,96,97,98,99,100,101,102,103,104,105,106,107].

Indeed, AI applications could direct the endoscopists in adopting a “diagnose-and-leave” strategy for hyperplastic polyps and a “resect-and-discard” strategy for diminutive colorectal adenomas, and hence reducing the healthcare costs associated with a colonoscopy [108]. To date, various methods have been developed and showed promising results for polyp characterization. In a recent study, Min et al. [101] designed a novel CADx system to predict the histology of colorectal polyps (adenomatous vs. non-adenomatous) by analyzing linked color imaging (LCI) images. The current algorithm demonstrated an accuracy of 78.4% (comparable to that of expert endoscopists) with 83.3% sensitivity, 70.1% specificity, 82.6% positive predictive value (PPV) and 71.2% NPV in distinguishing adenomatous from non-adenomatous polyps. At present, computer-aided models are also used to include information collected from dynamic fluorescence imaging during ongoing surgery to characterize the suspected lesions by their biology [109]. This intraprocedural approach could provide an analysis in a continuous fashion, which is more akin to real-life than an image-by-image analysis.

### 5.1. Magnification Endoscopy with Narrow-Band Imaging (NBI)

NBI, as an advanced endoscopic imaging modality, allows better visualization and assessment of the mucosal surface and microvascular patterns [110]. This technique is particularly essential in differentiating benign from premalignant lesions and evaluating the depth of submucosal infiltration [111,112,113]. Magnifying colonoscopy combined with NBI enables the endoscopists to improve the diagnostic accuracy of real-time optical diagnosis.

At first, Tischendorf et al. [114] designed and evaluated a computer-aided classification system on 128 patients with 209 colorectal polyps who underwent magnification endoscopy with NBI. However, the diagnostic accuracy of this algorithm (85.3%) was relatively lower than that of expert endoscopists. In 2011, Gross et al. [115] developed a computer-assisted model for polyp classification, which yielded a sensitivity, specificity and accuracy of 95%, 90.3%, and 93.1%, respectively. This algorithm was based on the analysis of nine selected vessel features, such as perimeter and brightness, from a total number of 214 patients with 434 colorectal polyps who underwent magnifying endoscopy with NBI. The results of this classification system were comparable to those of expert endoscopists (sensitivity, specificity and accuracy of 93.4%, 91.8%, and 92.7%, respectively) and significantly superior to those of novice endoscopists (sensitivity, specificity and accuracy of 86%, 87.8%, and 86.8% respectively). Concerning polyp characterization, research teams at Hiroshima University in Japan created novel CADx systems to predict neoplastic vs. non-neoplastic colorectal polyps with accurate and consistent diagnostic performance, suggesting that even real-time histological diagnosis is achievable as well [93,111,116,117,118,119,120].

Interestingly, Chen et al. [98] designed a deep learning model to classify diminutive colorectal polyps, which yielded a sensitivity of 96.3%, a specificity of 78.1% with an accuracy of 90.1%, PPV of 89.6%, and NPV of 91.5%. A sample of magnifying NBI images with 284 diminutive colorectal polyps extracted from a total number of 193 patients was tested to evaluate the diagnostic accuracy of the current computer-aided system. This algorithm was able to distinguish neoplastic from hyperplastic lesions in a shorter time compared to the time required by expert and trainee endoscopists (0.45 ± 0.07 s vs. 1.54 ± 1.30 s vs. 1.77 ± 1.37 s). In 2019, Byrne et al. [100] tested their deep CNN model on histology prediction by using 125 unaltered videos of colorectal polyps during a routine colonoscopy. Even though the algorithm did not generate sufficient confidence to predict the histological diagnosis of 19 diminutive colorectal polyps in the test set, the system showed 98% sensitivity, 83% specificity, 94% accuracy, 90% PPV and 97% NPV for the remaining 106 diminutive polyps.

### 5.2. Magnifying Chromoendoscopy

Magnifying chromoendoscopy represents a time-efficient technique, which allows the optical diagnosis of colorectal lesions, commonly resulting in high diagnostic performance (97.8% sensitivity, 91.4% specificity, and 97.1% accuracy when performed by experts) [121]. In this procedure, dye spray (indigo carmine or crystal violet) is used along with a high-resolution magnifying colonoscope to improve the inspection and analysis of pit patterns of the polyp surface. With the intent of improving the diagnostic accuracy in histology prediction of colorectal lesions, Häfner et al. [122] proposed an algorithm based on texture feature extraction approaches in the wavelet-domain. In another study, Takemura et al. [123] created a software model based on quantitative analysis of pit patterns for the differential diagnosis of colorectal lesions. Overall, texture and quantitative analysis (such as area, perimeter and circularity) of pit patterns represent two major techniques commonly used in automated computer-aided systems.

### 5.3. Endocytoscopy

Endocytoscopy constitutes an emerging endoscopic imaging modality, which allows in vivo microscopic imaging and real-time diagnosis of cellular structures at particularly high magnification (up to 400-fold or even up to 1400-fold magnification power in endoscope-based or probe-based endocytoscopy, respectively) during ongoing colonoscopy [124]. Based on the principle of contact light microscopy, this procedure enables the inspection of the superficial mucosal layer after the preparation and prestaining of the colonic mucosa with absorptive contrast agents, such as toluidine blue [124,125]. The application of such endoscopic techniques in clinical practice may lead to easier robust endocytoscopic image analysis using computer-aided software.

A Japanese research group conducted studies on newly designed computer-aided algorithms for in vivo histological differentiation of colorectal lesions using an endocytoscopy [126]. At first, they adjusted their model based on six nuclear features (area, standard deviation of area, circularity, circularity of the 20 largest nuclei, shortest and longest diameter) after nuclear segmentation from the endocytoscopic images. The present system demonstrated 92% sensitivity and 89.2% accuracy in establishing a precise diagnosis of polyp pathology. In the next few years, this research team enhanced their software algorithm by extracting features from texture analysis and utilizing SVM as a classifier for benign, adenomatous lesions or invasive carcinoma [95,127]. These researchers also developed another computer-assisted model based on a combination of endocytoscopy and NBI without using any dye solutions [94]. In fact, they assessed the microvessel findings from the polyp surface, showing an overall accuracy of 90%. Similarly, Takeda et al. [96] investigated the role of a computer-aided endocytoscopy system on the optical diagnosis of invasive colorectal carcinoma, and their algorithm yielded 89.4% sensitivity, 98.9% specificity, 94.1% accuracy, 98.8% PPV, and 90.1% NPV.

### 5.4. Confocal Laser Endomicroscopy

Confocal laser endomicroscopy, which represents a microscopic imaging modality, enables in vivo observation of cellular and subcellular structures (up to 250 μm in depth) at 1000-fold magnification power [128]. Based on the k-nearest neighbor classification, Andre et al. [129] suggested using an automated polyp characterization system, which showed an accuracy of 89.6% in distinguishing malignant from benign lesions. In another research work based on neural network analysis, Ştefănescu et al. [130] developed a diagnostic algorithm using confocal laser endomicroscopy images with an accuracy of 84.5% in the differentiation of advanced colorectal adenocarcinomas from the normal intestinal mucosa. However, the evaluation of the diagnostic performance of this procedure requires further assessment with randomized controlled trials.

### 5.5. Laser-Induced Fluorescence Spectroscopy (LIFS)

Laser-induced fluorescence spectroscopy (LIFS) is a technique carried out to provide a real-time prediction of lesion pathology by analyzing in vivo fluorescence emission from the targeted tissue, either healthy or neoplastic. At first, using LIFS, Kuiper et al. [131] conducted a study to assess the diagnostic performance of WavSTAT (Spectrascience Inc., San Diego, CA, USA), an optical biopsy device that is incorporated into a standard biopsy forceps. However, the diagnostic accuracy of the WavSTAT system alone and the algorithm combining a high-resolution endoscopy with WavSTAT proved to be insufficient for in vivo optical diagnosis of small lesions. In 2016, Rath et al. [132] evaluated LIFS using the new WavSTAT4 system for real-time in vivo prediction of colorectal polyp histology. Their system was tested on 137 diminutive polyps from 27 patients during screening or surveillance colonoscopy and achieved an overall accuracy of 84.7% with 81.8% sensitivity, 85.2% specificity, and 96.1% NPV. This novel technology allows accurate real-time differentiation of colorectal lesions reducing the costs and risks associated with diminutive polyps’ resection.

### 5.6. Autofluorescence Endoscopy (AFE)

The autofluorescence imaging (AFI) endoscope could be used for colorectal polyp characterization, analyzing the color differences in fluorescence emission of the tissue (emerging from endogenous fluorophores such as collagen and flavins) after the exposure and excitation by a light source. Using color analysis models may significantly contribute to the differentiation of non-neoplastic from neoplastic lesions during a colonoscopy examination based on AFI. In Japan, research groups conducted studies to assess the performance of novel computer-aided software on numerical color analysis, calculating the green/red ratios of the image during AFE, with several promising results [133,134].

### 5.7. White Light Endoscopy (WLE)

White light endoscopy (WLE) represents an endoscopic modality that could be used in combination with computer-assisted models to discriminate between neoplastic and non-neoplastic lesions. In a recent study, Komeda et al. [97] applied a CNN system for polyp classification using WLE with only 75.1% accuracy. They support the assertion that the diagnostic performance of WLE is inferior to NBI or chromoendoscopy with or without magnification. Further investigation is required to assess the accuracy of WLE combined with computer-aided software for colorectal lesion characterization.

## 6. Treatment

### 6.1. Robotic-Assisted Surgery

Colorectal cancer treatment enters a new era with the contribution of robotic colorectal surgery, an advanced form of minimally invasive surgery. To date, the da Vinci System (available models: da Vinci Si, X, Xi, SP) represents the most widely used robotic surgical system globally. It enables surgeons to perform very delicate or even highly complex procedures using wristed instruments with seven degrees of freedom. Robot-assisted surgery offers not only significant benefits to the patients but also the surgeons. These advantages include shorter length of recovery and hospital stay, minimal scarring, smaller incisions, and a significant reduction in the risk of surgical site infections, postoperative pain and blood loss compared to traditional open surgery [135,136]. Computer-controlled devices allow the surgeons to operate with enhanced visual field, flexibility, dexterity, precision, and minimal fatigue. The da Vinci dual-console also allows integrated teaching and supervising and offers the potential to alter residents’ surgical training. The Senhance surgical robotic system (TransEnterix Surgical Inc., Morrisville, NC, USA) is a laparoscopy-based system that enables experienced laparoscopic surgeons to turn to more complex procedures. Hirano et al. suggested the use of the present system as a safe, effective and precise surgical treatment in patients with colon cancer [137].

Based on current research, when compared with open surgery for colorectal cancer, robotic surgery appeared to result in a less pronounced inflammatory response and lower complication and conversion rates [138,139]. Meanwhile, there is a plethora of literature suggesting that both robotic and laparoscopic approaches are equivalent in terms of perioperative outcomes for CRC patients [140]. However, previous studies showed enhanced postoperative recovery and better conversion rates regarding robot-assisted surgery for rectal cancer [141,142,143]. Park et al. [144] mentioned that the conversion rate in the laparoscopic vs. the robotic group was 7.1% vs. 0 (*p* = 0.003), respectively, when performed by an experienced operator. At this point, it is essential to understand that the safety and success of a particular surgical procedure significantly depend on the adequacy of surgeons’ training. So far, the learning curve for robotic colorectal surgery seems to be shorter than that required for conventional laparoscopic surgery [6].

The laparoscopic approach for rectal cancer resection is considered technically challenging in several cases, including male, obese patients or patients with difficult pelvic anatomy [145,146]. The robotic platform offers particular advantages by providing access to hard-to-reach areas, such as a narrow pelvis, and preserving the postoperative urinary and sexual function integrity [147]. Indeed, in the ROLARR randomized clinical trial, Jayne et al. reported that laparoscopic rectal surgery was associated with higher conversion rates in males, obese patients and patients undergoing a low anterior resection compared to robotic surgery [146]. Current studies also revealed that robotic-assisted surgery appears to be more suitable for pelvic autonomic nerve protection [147,148].

### 6.2. Chemotherapy

Based on computer-aided drug delivery techniques, Cruz et al. [149] developed a model to detect the half-maximal inhibitory concentration of a drug against the human colon carcinoma HCT116 cell line using molecular and nuclear magnetic resonance. The current method achieved an overall prediction accuracy of over 63% for both training and test sets. The improvement of docking-based virtual screening represents another challenging issue in drug discovery. Berishvili et al. [150] created a deep neural network algorithm to develop anticancer drugs that inhibit PI3K alpha (PI3Ka) and tankyrase, promising targets for CRC treatment. Novel techniques of tumour targeting focus on the use of nanoparticles as pharmaceutical carriers [151]. Alternatively, medical nanorobotic agents could be effective in cancer treatment by achieving an optimal targeting approach. Martel et al. [152] proposed using a computer-aided magnetotactic displacement technique to navigate and deliver the drug-loaded magnetotactic bacteria MC-1 towards the hypoxic areas of tumours.

With the intent of identifying the pathological complete responder (CR) and non-responder (NR) patients after neoadjuvant chemoradiotherapy (CRT) in locally advanced rectal cancer (LARC), Ferrari et al. [153] used the random forest algorithm to create two AI models. Analyzing the textural features of T2-weighted magnetic resonance (MR) images, the current models demonstrated an AUC of 0.86 and 0.83 for pathological CRs and NRs, respectively. In another research concerning patients with LARC, Shi et al. [154] constructed a CNN model to predict the neoadjuvant CRT response based on data collected from pre-treatment and early-treatment follow-up magnetic resonance imaging (MRI, 3–4 weeks after starting CRT). Abraham et al. [155] used a machine learning approach to identify a 67-gene signature (the “FOLFOXai” signature) predictive of the efficacy of oxaliplatin-based chemotherapy combined with bevacizumab in patients with metastatic colorectal carcinomas. Consequently, these AI applications may help physicians to provide more effective treatment strategies even at the early stages of CRT. Interestingly, using machine learning techniques, Oyaga-Iriarte et al. [156] conducted a study to predict whether metastatic CRC patients would suffer from high degrees of toxicity of a drug referred to as irinotecan (showing accuracy of 76%, 75% and 91% for leukopenia, neutropenia and diarrhea, respectively).

## 7. Current Status of Precision Oncology in Colorectal Cancer

The inherent tumour heterogeneity lends itself to the rapidly emerging field of precision medicine (or personalized medicine) [7]. Precision oncology (or personalized cancer medicine), a significant translational medicine paradigm, may assist physicians in guiding management decisions of cancer patients by achieving a more personalized and targeted therapy. This approach tailors treatment strategies to the individual patient considering each person’s variability in genes, environment, and lifestyle [157].

Based on genetically altered cancer genes that affect drug responses, precision oncology aims to provide more effective targeted treatment and new hope for stratifying therapeutic strategies. In 2019, a research team developed a machine learning-based algorithm to detect specific patient subpopulations that react in a different manner to the inhibitors of the same or different targets, simultaneously gaining a better understanding of the mechanisms of resistance and pathway cross-talk [158]. The current model may identify new cancer subpopulations, their genetic biomarkers, and drug combinations with improved efficacy [158].

S100A9, a calcium-binding protein, may represent a potential therapeutic target for CRC. Based on machine learning techniques, Lee et al. [159] developed an algorithm to predict the protein–protein interactions of S100A9 with drugs and then evaluated the drug specificity on 2D molecular descriptors. In another study, the authors designed an AI model to identify candidate molecular biomarkers for CRC by integrating transcriptomics and proteomics data at the system biology level [160]. Based on RNA-sequencing data, Pacheco et al. [161] constructed a metabolic model to identify those drugs that target cancer-specific metabolism.

Currently, a research group used machine learning techniques and specific phenotypic studies accompanied by mechanistic studies, chemical genetics, and omics assays to successfully predict disease-drug pairs with the intent of repurposing existing drugs for CRC treatment [162]. Furthermore, according to the data collected in the preoperative period, Horta et al. [163] designed a prediction model to postoperatively support clinical decisions for the co-management between internists and surgeons of the selected patients.

“Watson for Oncology” (WFO, IBM Corporation, United States) represents an AI-based system that may assist in the field of precision oncology. During clinical trials, nearly 90% of Watson’s treatment recommendations were in line with those of its human teachers [164]. In Japan, a research group implemented IBM Watson for the whole genome sequencing and analysis of cancer patients and produced results within only four days [165]. As all these revolutionary approaches to cancer patients’ care hold the key to effective treatment in the near future, we should move away step by step from the one-size-fits-all model. Hippocrates [166], who is regarded as the father of western medicine, almost 2500 years ago, advised: “give different [liquid medicines] to different patients, for the sweet ones do not benefit everyone, nor do the astringent ones, nor are all the patients able to drink the same things”. In summary, it is worth mentioning that the contribution of artificial intelligence and highly personalized therapeutic methods to cancer treatment may improve the clinical outcomes by providing “the right drug for the right patient at the right time [167]”.

## 8. Solutions, Limitations and Future Directions

AI provides computer-aided solutions with significant benefits for CRC patients. Advances in deep learning techniques paved the way to enhance our understanding of colorectal tumorigenesis and cancer progression. Novel methods assist doctors in detecting and diagnosing premalignant and malignant lesions based on images and biopsy samples. AI algorithms are used to develop targeted treatment strategies for the patients promoting the personalization of CRC management. In fact, these technological advancements improve the speed and accuracy of diagnoses. Computer-aided systems are not subject to distraction or fatigue, and intra-observer variation.

To date, AI tools can at least match or even exceed human performance for CRC detection and diagnosis [168]. CADe models may act as a “second observer” during colonoscopy procedures and become useful for junior endoscopists’ training [71]. AI systems are also used for quality assessment of screening and diagnostic procedures. Indeed, based on natural language processing (NLP), researchers designed and tested a model across multiple institutions to measure colonoscopy quality [169]. Most AI-powered virtual assistants provide personalized healthcare services and improve the communication between patients and care providers. In addition, AI-based applications in mobile devices, such as the Colorectal Cancer Awareness Application (ColorApp), may be used to improve community education and participation in CRC screening programs [170].

Despite the significant benefits of AI applications in CRC diagnosis and treatment, the development of AI-based technology faces several limitations. Indeed, the ability to train a machine to “think” like a human being is a complex task, and its success depends on many factors. First, the difficulty of integrating this advanced technology into clinical workflows and large-scale implementation is considered an essential obstacle in AI progress. As new approvals of these novel methods become available by governmental and professional organizations, it will become even easier to use AI tools for standard patient care in daily practice. In addition to obstacles for the Food and Drug Administration (FDA) approval, developing AI models is considered a notoriously expensive process [171].

Furthermore, without sufficient high-quality training data and robust computational infrastructure, even the most state-of-the-art algorithms are doomed to failure [172]. In general, deep learning algorithms require large volumes of data to train the AI systems at the best level to obtain the best results. Despite the efforts to standardize patient care, the variability between patients concerning differences in clinical presentation and desired outcomes should be thoroughly considered prior to AI model development [173]. Any potential deviation from the training conditions may result in the unpredictable behavior of an algorithm [172].

While we know the input and output data an algorithm produces, there is limited information about its internal workings and processes. This issue is referred to as the “black box” problem in machine learning [172]. The “black box” problem indicates that significant confounding from any of the many factors could easily be missed, especially in deep learning algorithms with many hidden layers (e.g., classifying skin lesions as malignant or benign [174]). When there is limited visibility into understanding how the algorithm has made a particular decision, evaluating its safety and suitability in patients is questionable [172]. The use of AI models in common practice raises a whole set of ethical and legal issues. Who has liability when an algorithm makes an error with severe consequences? Is that just the hospital or the doctor who applied it in a certain way? Is it the manufacturer or the regulator of the algorithm who approved it? Any expressed concern about AI applications should be addressed before their implementation. Professional organizations should also establish guidelines to promote the trustworthy development and application of AI systems in healthcare.

Evaluating the AI algorithms’ performance requires further validation with randomized controlled trials beyond the original clinical centers of development prior to widespread clinical implementation [171]. This novel technology also faces other challenging issues regarding privacy protection. Eventual relationships between healthcare companies and academic research data may elevate the risk of malicious privacy violations [175].

However, the medical community remains highly optimistic about the future of AI healthcare applications. Machine learning techniques will not replace doctors, but they can significantly contribute to CRC screening, diagnosis, and treatment. AI can enhance clinical practice and provide essential improvements in several areas of interest associated with colorectal tumours.

The integration of AI-based platforms that can “read” data from a colonoscopy video or the existing electronic medical records (EMR) enables the systems to use this information either for training or real-time decision support [176]. Big data represent massive amounts of information that is unmanageable using traditional software. They can be classified by 5 Vs; volume, velocity, variety, veracity, and value [177]. In recent years, deep learning methods assist doctors and researchers in processing a large number of databases and discovering the hidden opportunities in data [178]. AI techniques help doctors analyze the patient’s medical history and provide the best treatment options. Consequently, this advanced technology has a promising future in processing and handling big data. Shortly, further research will be required to design the appropriate security and privacy measures to protect and manage medical data safely.

As discussed in this review, the AI systems are expected to improve polyp detection and characterization. Prospective studies with real-time use of computer-aided systems are necessary to ensure the replicability or repeatability of the results and incorporate this novel technology into clinical practice. In addition, it is vital to investigate AI applications’ role in detecting and diagnosing lesions with malignant potential (e.g., sessile serrated lesions, colitis-associated cancer).

## 9. Conclusions

Overall, the integration of AI applications in screening, diagnosis, and treatment of CRC may improve clinical outcomes and prognosis for the patients. In recent years, deep learning techniques are further applied in clinical cancer research. A key challenge in clinical practice is also the development of optimal therapies, which would provide novel targeted approaches or alternative therapeutic options. Our research work reveals the necessity to deeply understand the challenges and opportunities presented by AI-based models in the fields of CRC screening, diagnosis, and patient care. At the same time, AI is considered a valuable tool in transforming the future of healthcare and precision oncology. Computer-aided systems can provide physicians with assistance in detecting and diagnosing precancerous lesions or early-stage CRC. Several novel algorithms have shown promising results for the accurate detection and characterization of suspected lesions. However, additional prospective, large-scale, multicenter clinical trials are required to evaluate the diagnostic accuracy of AI systems.

## Figures and Tables

**Table 1:** Summary of recent studies on AI systems for colorectal polyp detection.

Author, Year	Country	Study Design	AI Algorithm	Type of Images	Outcomes
Fernández-Esparrach et al., 2016 [71]	Spain	Retrospective	WM-DOVA energy maps	24 videos containing 31 colorectal polyps	Sensitivity: 70.4% Specificity: 72.4%
Geetha et al., 2016 [72]	India	Ex vivo	Hand crafted	Still images, 703 frames	Sensitivity: 95% Specificity: 97%
Yu et al., 2017 [73]	China	Ex vivo	CNN	Videos, ASU-Mayo 18 colonoscopy videos	Sensitivity: 71% PPV: 88%
Zhang et al., 2017 [74]	China	Ex vivo	CNN	Still images	Accuracy: 86% AUC: 1
Billah et al., 2017 [75]	Bangladesh	Ex vivo	CNN	14,000 still images	Sensitivity: 99% Specificity: 99% Accuracy: 99%
Misawa et al., 2018 [76]	Japan	Ex vivo	CNN	Videos	Per-frame sensitivity: 90% Specificity: 63.3% Accuracy: 76.5% Per-polyp sensitivity: 94% False positive rate: 60%
Urban et al., 2018 [77]	United States	Ex vivo	CNN	Videos	Sensitivity: 90%
Figueiredo et al., 2019 [78]	Portugal	Retrospective	SVM binary classifiers	42 colonoscopy videos containing 1680 frames with polyps and 1360 frames without polyps	Sensitivity: 99.7% Specificity: 84.9% Accuracy: 91.1%
Klare et al., 2019 [79]	Germany	In vivo, prospective cohort	KoloPol software	Real-time colonoscopy	Per-polyp sensitivity: 75% ADR in CADe group vs colonoscopy group: 29% vs. 31%
Yamada et al., 2019 [80]	Japan	Ex vivo	CNN	Videos	Sensitivity: 97.3% Specificity: 99% AUC: 0.975
Wang et al., 2019 [48]	China	Prospective, RCT	EndoScreener	Real-time colonoscopy	ADR in CADe group vs standard colonoscopy group: 29.1% vs. 20.3%, *p* < 0.001
Liu et al., 2020 [81]	China	Prospective, RCT	Henan Tongyu	Real-time colonoscopy	ADR in CADe group vs control group: 39.2% vs. 24%
Su et al., 2020 [82]	China	Prospective, RCT	Deep CNNs	Real-time colonoscopy	ADR in CADe group vs control group: 28.9% vs. 16.5%
Ozawa et al., 2020 [83]	Japan	Ex vivo	CNN	7077 images	Sensitivity: 92% Accuracy: 83% PPV: 86%
Gong et al., 2020 [84]	China	Prospective, RCT	ENDOANGEL	Real-time colonoscopy	ADR in CADe group vs. control group: 16% vs. 8%
Wang et al., 2020 [85]	China	Double-blind, RCT	EndoScreener	Real-time colonoscopy	ADR in CADe group (484 patients) vs control group (478 patients): 34.1% vs. 28%
Hassan et al., 2020 [86]	Italy	Retrospective	GI Genius	338 videos	Per-lesion sensitivity: 99.7%
Repici et al., 2020 [87]	Italy	RCT	GI Genius	Real-time colonoscopy	ADR in CADe group vs. control group: 54.8% vs. 40.4%

AI: artificial intelligence; WM-DOVA: Window Median Depth of Valleys Accumulation; CNN: convolutional neural network; PPV: positive predictive value; AUC: area under the curve; SVM: support vector machine; ADR: adenoma detection rate; CADe: computer-aided detection; RCT: randomized controlled trial.

**Table 2:** Summary of recent studies on AI systems for colorectal polyp characterization.

Author, Year	Country	Study Type	Patients/ Polyps	Imaging Modality	AI Algorithm	Real-Time	Outcomes	Notes
Kominami et al., 2016 [93]	Japan	Prospective	41/118	Magnifying NBI	SVM	Yes	Sensitivity: 93% Specificity: 93.3% Accuracy: 93.2% PPV: 93% NPV: 93.3%	Diminutive polyps were involved. Endoscopists were used as controls. Histologic findings were used as the reference standard
Misawa et al., 2016 [94]	Japan	Retrospective	NA/100	Endocytoscopy with NBI	EndoBRAIN	No	Sensitivity: 84.5% Specificity: 97.6% Accuracy: 90% PPV: 98%, NPV: 82%	Histologic findings were used as the reference standard
Mori et al., 2016 [95]	Japan	Retrospective	123/205	Endocytoscopy	SVM	No	Sensitivity: 89% Specificity: 88% Accuracy: 89% PPV: 95% NPV: 76%	Diminutive polyps were involved. Endoscopists were used as controls. Histologic findings were used as the reference standard
Takeda et al., 2017 [96]	Japan	Retrospective	76/76	Endocytoscopy	SVM	No	Sensitivity: 89.4% Specificity: 98.9% Accuracy: 94.1% PPV: 98.8% NPV: 90.1%	CADx system for differentiation between invasive CRC and adenomatous polyps. Histologic findings were used as the reference standard
Komeda et al., 2017 [97]	Japan	Retrospective	NA/NA	A combination of WLE, NBI and Chromoendoscopy	CNN	Yes	Accuracy: 75.1%	Histologic findings were used as the reference standard
Chen et al., 2018 [98]	Taiwan	Prospective	193/284	Magnifying NBI	CNN	No (real-time capability)	Sensitivity: 96.3% Specificity: 78.1% Accuracy: 90.1% PPV: 89.6% NPV: 91.5%	Diminutive polyps were involved. Endoscopists were used as controls. Histologic findings were used as the reference standard
Mori et al., 2018 [49]	Japan	Prospective	325/466	Endocytoscopy with NBI and ΜΒ staining modes	SVM	Yes	Sensitivity: >90% Specificity: ~70% for identifying proximal diminutive adenomas. Accuracy: 98.1% NPV: 96.4%	Diminutive polyps were involved. Endoscopists were used as controls. Histologic findings were used as the reference standard
Renner et al., 2018 [99]	Germany	Retrospective	NA/100	WLE, NBI	Deep neural network	No	Sensitivity: 92.3% Specificity: 62.5% Accuracy: 78% PPV: 72.7% NPV: 88.2%	Diminutive polyps were involved. Endoscopists were used as controls. Histologic findings were used as the reference standard
Byrne et al., 2019 [100]	Canada	Retrospective	NA/106	NBI	CNN	Real-time capability	Sensitivity: 98% Specificity: 83% Accuracy: 94% PPV: 90% NPV: 97%	Diminutive polyps were involved. Histologic findings were used as the reference standard
Min et al., 2019 [101]	China	Prospective	91/181	LCI	Gaussian mixture model	No	Sensitivity: 83.3% Specificity: 70.1% Accuracy: 78.4% PPV: 82.6% NPV: 71.2%	Endoscopists were used as controls. Histologic findings were used as the reference standard
Sánchez-Montes et al., 2019 [102]	Spain	Retrospective	NA/225	WLE	SVMs	No	Sensitivity: 92.3% Specificity: 89.2% Accuracy: 91.1% PPV: 93.6% NPV: 87.1%	Diminutive polyps were involved. Endoscopists were used as controls. Histologic findings were used as the reference standard
Lui et al., 2019 [103]	China	Retrospective	NA/76	WLE, NBI	CNN	No	Sensitivity: 88.2% Specificity: 77.9% Accuracy: 85.5%	CADx system for invasive CRC diagnosis. Endoscopists were used as controls. Histologic findings were used as the reference standard
Horiuchi et al., 2019 [104]	Japan	Prospective	95/429	AFI	Color intensity analysis software	Yes	Sensitivity: 80% Specificity: 95.3% Accuracy: 91.5% PPV: 85.2% NPV: 93.4%	Diminutive polyps were involved. Endoscopists were used as controls. Histologic findings were used as the reference standard
Ozawa et al., 2020 [83]	Japan	Retrospective	174/309	NBI	CNN	No (real-time capability)	Sensitivity: 97% PPV: 84% NPV: 88%	AI system for characterization and detection of colorectal polyps. Diminutive polyps were involved. Endoscopists were used as controls. Histologic findings were used as the reference standard
Rodriguez-Diaz et al., 2020 [105]	United States	Prospective	119/280	Magnifying NBI	DL, a semantic segmentation model based on DeepLab V3+ framework with ResNet18-based feature extractor	Yes	Sensitivity: 96% Specificity: 84% NPV: 91%, HCR: 88% For diminutive colorectal polyps: Sensitivity: 95% Specificity: 88% NPV: 93%, HCR: 86%	Diminutive polyps were involved. Endoscopists were used as controls. Histologic findings were used as the reference standard
Jin et al., 2020 [106]	South Korea	Prospective	NA/300	NBI	CNN	No	Sensitivity: 83.3% Specificity: 91.7% Accuracy: 86.7% PPV: 93.8% NPV: 78.6%	Diminutive polyps were involved. Endoscopists were used as controls. Histologic findings were used as the reference standard
Kudo et al., 2020 [107]	Japan	Retrospective	NA/2000	Endocytoscopy with NBI and ΜΒ staining modes	EndoBRAIN	No	NBI Sensitivity: 96.9% Specificity: 94.3% Accuracy: 96% PPV: 96.9% NPV: 94.3% Stained images Sensitivity: 96.9% Specificity: 100% Accuracy: 98% PPV: 100% NPV: 94.6%	Diminutive polyps were involved. Endoscopists were used as controls. Histologic findings were used as the reference standard

AI: artificial intelligence; NBI: narrow-band imaging; SVM: support vector machine; PPV: positive predictive value; NPV: negative predictive value; NA: not available; CADx: computer-aided diagnosis; CRC: colorectal cancer; WLE: white light endoscopy; CNN: convolutional neural network; MB: methylene blue; LCI: linked color imaging; AFI: autofluorescence imaging; DL: deep learning; HCR: high-confidence rate.
